# Construction of a core collection of native *Perilla* germplasm collected from South Korea based on SSR markers and morphological characteristics

**DOI:** 10.1038/s41598-021-03362-0

**Published:** 2021-12-13

**Authors:** Kyu Jin Sa, Dong Min Kim, Jun Seok Oh, Hyeon Park, Do Yoon Hyun, Sookyeong Lee, Ju Hee Rhee, Ju Kyong Lee

**Affiliations:** 1 Department of Applied Plant Sciences, College of Agriculture and Life Sciences, Kangwon National University, Chuncheon, 24341 Korea; 2 Interdisciplinary Program in Smart Agriculture, Kangwon National University, Chuncheon, 24341 Korea; 3 National Agrobiodiversity Center, National Institute of Agricultural Sciences, RDA, Jeonju, 54874 Korea; 4 Korea National College of Agriculture and Fisheries, Jeonju, 54874 Korea

**Keywords:** Genetics, Plant sciences

## Abstract

The leaves and seed oil of *Perilla* crop (*Perilla frutescens* L.) have attracted interest as health foods in East Asia. This crop has been traditionally cultivated and used for a long time as a folk plant, especially in Korea. In our study, the 22 SSR markers and eight morphological traits were used to assess the genetic diversity and population structure, to select a core collection of 400 *Perilla* accessions conserved in the RDA-Genebank of South Korea. A total of 173 alleles were detected and the number of alleles per locus ranged from 4 to 15 (average = 7.9). Gene diversity and polymorphic information content ranged from 0.138 to 0.868 (average = 0.567) and 0.134 to 0.853 (average = 0.522), respectively. The 400 accessions were not clearly distinguished geographically by STRUCTURE and UPGMA analyses. A core collection (44 accessions) was selected from the entire collection by using PowerCore. The core collection accounted for 11.0% of the entire *Perilla* collection, including 100% of the number of alleles maintained in the whole collection and with similar or greater Shannon–Weaver and Nei diversity indices than the whole collection. The core collection selected by SSR markers was evenly distributed in three clusters on a scatter plot by eight morphological traits. The first core collection of *Perilla* accessions was constructed, and it maintained allelic richness. Further modification of the core collection is expected with the continuous addition of new accessions of the two cultivated types of *Perilla* crop and their weedy types.

## Introduction

Plant genetic resources (PGRs) mean individuals or populations of wild species, genetic stocks, and cultivars, which are maintained as types of plants, seeds, and tissues^1^. These PGRs are utilized to provide new plant varieties with better resistance, adaptation, and nutritive qualities. Conservation and collection of PGRs is necessary to maintain higher genetic diversity for food security and use in the future^2^. Moreover, the conservation and collection of PGRs in most crops has been made possible by the establishment of genebanks in countries around the world. The fundamental role of a genebank is to increase genetic diversity and prevent genetic erosion by collecting, conserving, and documenting PGRs. In South Korea, the Genebank of the National Agrobiodiversity Center (NAAS) of the Rural Development Administration (RDA-Genebank) plays an important role in supporting genetic resource conservation and utilization. The RDA-Genebank currently preserves a total of 266,649 genetic resources for 3,083 species, including food, horticulture, industrial, and forage crops (http://genebank.rda.go.kr). Although a large number of PGRs have been collected in genebanks, the large sample size and lack of adequate information about population structure and genetic diversity of these collections hinders the successful utilization of the genetic potential for PGRs^3^. Efficient management and utilization of the existing collections could be greatly enhanced by reducing the number of accessions from large collections^4^. Frankel (1984) suggested the concept of a core collection, which is a subset of accessions with minimum redundancy and maximum genetic diversity from an entire collection. Various studies have proposed subsampling proportions ranging from 5 to 30% for a core collection^3,6–8^. A core collection is commonly established to conserve phenotypic and genetic diversity and enable the selection of candidate alleles associated with important agronomic traits^9^. Development of a core collections has been promoted as the most important activity in conservation and utilization of PGRs since 1996^2^. Therefore, core collections have already been reported for many crops, such as rice^10^, soybean^11^, and wheat^12^. The development of core collections was based at first on passport data, geographical distribution, and phenotypic data^13–15^. Subsequently, the advance of molecular markers, which are an efficient means of confirming genetic diversity, has allowed the development of more powerful core collections in many crop species, either alone^16^ or in combination with phenotypic data^4,17^. Among various molecular markers, simple sequence repeats (SSRs) are often selected for genetic studies, such as for genetic diversity and the development of core collections, because of their advantages such as high reproducibility, polymorphism, abundance, and codominance^12,18,19^. Although there are a number of useful methods for selection of a core collection, the power core method for the development of a core collection utilizes the advanced M (maximization) strategy implemented through a modified heuristic algorithm by Kim et al. (2007). Using this program, core collections have been developed in various crops, such as rice^21^, soybean^11^, barnyard millet^22^, ragi^23^, and cassava^24^.

*Perilla* crop (*Perilla frutescens* L., 2n = 40) is an annual self-fertilizing species of the Lamiaceae family. This species has been traditionally cultivated and used for a long time as a folk plant, especially in East Asian countries such as Korea, Japan, and China. It is divided into two different varieties (or cultivated types), *P. frutescens* var. *frutescens* and var. *crispa*, based on morphology and use in East Asia. For example, *P*. *frutescens* var. *frutescens,* which is called “dlggae” in Korea, “egoma” in Japan, and “ren” in China, is tall with a large seed size (more than 2 mm), green leaf and stem color, and non-wrinkled leaf and used as an oil and leafy vegetable crop^25,26^. In contrast, *P*. *frutescens* var. *crispa,* which is called “cha-jo-ki” in Korea, “shiso” in Japan, and “zisu” in China, is short with a small seed size (less than 2 mm), red or green leaves and stems, and wrinkly or non-wrinkly leaves and is used as a vegetable and herbal medicine crop^25,26^.

*P. frutescens* var. *frutescens* has become a cash crop in South Korea. The leaves of var. *frutescens* are abundant in vitamin B and C, and it is a favored salad vegetable eaten with meat and used for pickles. Furthermore, *Perilla* seed oil of cultivated *P. frutescens* var. *frutescens* has a high content of polyunsaturated fatty acids, such as linoleic acid (C18:2) and α-linolenic acid (C18:3), which comprise approximately 80% of *Perilla* seed oil. The *Perilla* seed oil, similarly to soybean, rapeseed, corn, and sesame seed oils, has been used for foods such as cooking oils^27,28^. Recently, the leaves and seed oil have attracted interest as health foods in South Korea; therefore, the cultivation area of *P. frutescens* var. *frutescens* has increased greatly. Although approximately thirty commercial varieties have been registered in Korea (http://www.seed.go.kr), there have been few efforts to develop new cultivars for *Perilla* crop. Most farmers still cultivate landraces of their own regions^29^. The RDA-Genebank in South Korea maintains 2,368 genetic resources of cultivated and weedy types of *Perilla* crop, which are available to researchers and farmers (http://www.genebank.go.kr/).

To maximize the utilization of *Perilla* resources and for more efficient breeding programs, the morphological and genetic characteristics of the collected resources should be defined before use. Although many *Perilla* accessions in the RDA-Genebank have already been evaluated for phenotypic characteristics and diversity in the field, these characteristics are easily affected by environmental effects and evaluations are time and labor-intensive^30^. To overcome these limitations, molecular marker systems could be used to mitigate against the environmental effects and provide a better explanation of the variation than phenotype-based evaluation^31^. However, there has been a lack of information about genetic diversity and relationships at the molecular level for the RDA-Genebank accessions. Most of the *Perilla* accessions of the RDA-Genebank have been used rarely or not at all in *Perilla* crop breeding programs. Moreover, there are no reports of a core collection of *Perilla* based on DNA molecular markers.

Therefore, in our study, we performed population structure and genetic diversity analyses of 400 accessions of the *Perilla* germplasm collection of the RDA-Genebank using 22 *Perilla* SSRs. This allowed us to 1) examine the level of genetic diversity and the population structure within accessions of cultivated *P*. *frutescens* var. *frutescens* of the RDA-Genebank and 2) select a *Perilla* core collection that represents the entire collection without redundancy. This core group will provide useful information for efficient conservation and the utilization of genetic resources as well as for the selection of useful genetic resources for *Perilla* breeding programs.

## Results

### Genetic variation in the whole collection determined using SSR markers

A total of 22 SSR loci were used to evaluate the GDI, population structure, and genetic relationships among the 400 accessions of cultivated *P*. *frutescens* var. *frutescens* (Table 1). In this study, the 22 SSR primer sets were selected in a preliminary study using about 120 SSR primer sets to identify polymorphic SSR primer sets. Finally, a total of 22 SSR loci were confirmed within 173 alleles in the 400 accessions. The number of alleles per locus ranged from 4 (KNUPF36, KNUPF37, KNUPF59, KNUPF74) to 15 (KNUPF89), and the average number of alleles per locus was 7.9 (Table 1). The average GD was 0.567, with a range from 0.138 (KNUPF59) to 0.868 (KNUPF10). The average PIC value was 0.522, with a range of 0.134 (KNUPF59) – 0.853 (KNUPF10). The average MAF was 0.566, with a range of 0.195 (KNUPF10) – 0.928 (KNUPF59) (Table 1).

**Table 1:** Characteristics of the 22 SSR loci including allele number and GDI among 400 accessions of cultivated type of var. *frutescens*.

SSR loci	No. of Allele	GD	PIC	MAF
KNUPF1	6	0.387	0.367	0.773
KNUPF2	10	0.692	0.640	0.425
KNUPF4	5	0.676	0.620	0.430
KNUPF5	7	0.664	0.606	0.450
KNUPF10	14	0.868	0.853	0.195
KNUPF16	10	0.744	0.707	0.385
KNUPF23	5	0.384	0.332	0.755
KNUPF25	12	0.549	0.505	0.630
KNUPF26	5	0.355	0.307	0.778
KNUPF28	9	0.408	0.374	0.750
KNUPF36	4	0.612	0.544	0.523
KNUPF37	4	0.481	0.414	0.665
KNUPF39	11	0.767	0.736	0.380
KNUPF43	7	0.416	0.373	0.738
KNUPF55	10	0.688	0.648	0.480
KNUPF59	4	0.138	0.134	0.928
KNUPF71	4	0.521	0.409	0.528
KNUPF74	4	0.357	0.328	0.788
KNUPF77	8	0.516	0.422	0.590
KNUPF89	15	0.813	0.795	0.355
KNUPF107	6	0.608	0.576	0.590
KNUPF113	13	0.822	0.803	0.318
Total	173			
Mean	7.86	0.567	0.522	0.566

GD: gene diversity, PIC: polymorphic information content, MAF: major allele frequency.

**Figure 1:**
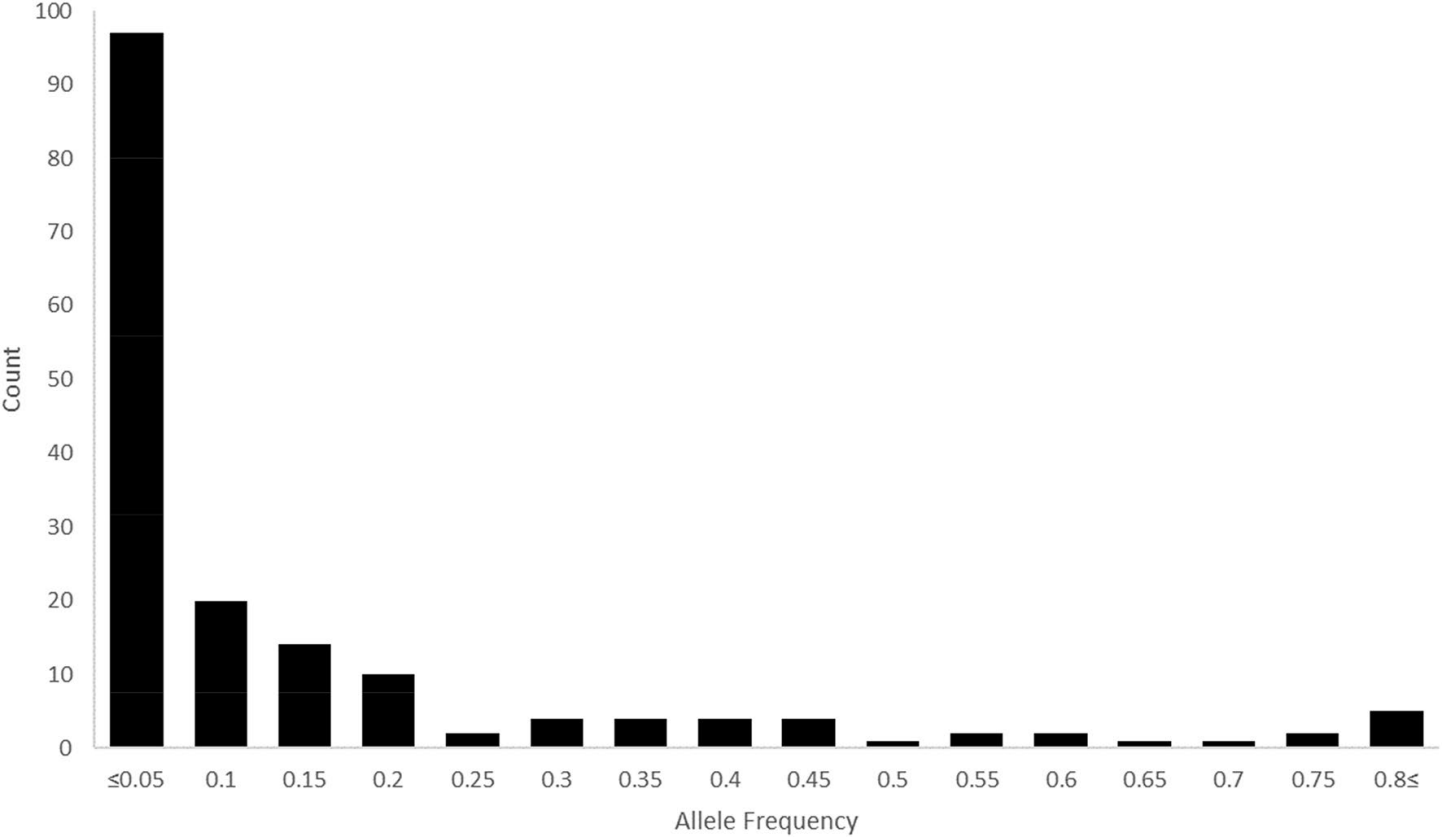
Histogram of allele frequencies in 400 accessions of cultivated type of var. *frutescens* based on 22 SSR markers.

To confirm the geographical difference for genetic diversity for accessions collected from central (Group I, 148 accessions) and southern (Group II, 211 accessions) regions of South Korea and foreign or unknown (Group III, 41 accessions), our study compared the number of alleles and the GDI of the three groups (Table 2). The average number of alleles was 6.5, 6.9, and 5.1 alleles for accessions of Group I, II, and III, respectively. In the results for GDI, the average GD values were 0.562, 0.554, and 0.578 for Group I, II, and III, respectively. The average PIC values were 0.521, 0.506, and 0.533 for Group I, II, and III, respectively. The average MAF values was 0.576, 0.571, and 0.541 for Group I, II, and III, respectively (Table 2). Among the 173 alleles, 23 private alleles (13.3%) were only detected in one of the 400 accessions of cultivated *P*. *frutescens* var. *frutescens*. The percentage of rare alleles (frequency < 0.05) was 56.1% (97 alleles) among the 173 alleles, whereas intermediate-frequency alleles (frequency of 0.05–0.5) and abundant alleles (frequency > 0.5) represented 36.4% (63 alleles) and 7.5% (13 alleles), respectively, of the 173 alleles (Fig. 1).

**Table 2:** Comparison of genetic diversity index between accessions collected from different regions using 22 SSR markers.

SSR loci	No. of Allele			GD			PIC			MAF		
	Group I (n = 148)	Group II (n = 211)	Group III (n = 41)	Group I (n = 148)	Group II (n = 211)	Group III (n = 41)	Group I (n = 148)	Group II (n = 211)	Group III (n = 41)	Group I (n = 148)	Group II (n = 211)	Group III (n = 41)
KNUPF1	4	6	5	0.464	0.266	0.591	0.432	0.256	0.544	0.709	0.853	0.585
KNUPF2	8	9	4	0.719	0.667	0.666	0.671	0.612	0.616	0.372	0.450	0.488
KNUPF4	5	5	5	0.702	0.628	0.764	0.652	0.560	0.725	0.405	0.474	0.293
KNUPF5	6	6	6	0.678	0.645	0.651	0.625	0.583	0.596	0.453	0.474	0.488
KNUPF10	12	13	9	0.846	0.871	0.857	0.828	0.858	0.840	0.230	0.209	0.195
KNUPF16	7	10	6	0.747	0.746	0.704	0.712	0.706	0.659	0.399	0.365	0.439
KNUPF23	4	5	3	0.417	0.360	0.380	0.355	0.316	0.324	0.723	0.777	0.756
KNUPF25	10	5	6	0.536	0.513	0.672	0.498	0.471	0.612	0.649	0.664	0.390
KNUPF26	5	3	4	0.390	0.350	0.222	0.337	0.295	0.212	0.750	0.777	0.878
KNUPF28	7	6	5	0.373	0.390	0.539	0.357	0.349	0.457	0.784	0.758	0.585
KNUPF36	4	4	3	0.561	0.623	0.608	0.503	0.548	0.534	0.601	0.469	0.512
KNUPF37	4	4	4	0.442	0.485	0.565	0.387	0.402	0.517	0.709	0.645	0.610
KNUPF39	8	9	7	0.764	0.757	0.742	0.732	0.724	0.708	0.378	0.374	0.415
KNUPF43	4	6	4	0.315	0.455	0.501	0.293	0.397	0.443	0.818	0.697	0.659
KNUPF55	9	9	6	0.652	0.659	0.772	0.622	0.601	0.739	0.547	0.460	0.341
KNUPF59	4	3	2	0.165	0.126	0.093	0.161	0.122	0.088	0.912	0.934	0.951
KNUPF71	3	4	3	0.500	0.526	0.504	0.394	0.415	0.400	0.588	0.512	0.585
KNUPF74	3	4	2	0.414	0.362	0.048	0.376	0.330	0.046	0.743	0.782	0.976
KNUPF77	5	7	3	0.495	0.505	0.509	0.412	0.412	0.420	0.635	0.607	0.610
KNUPF89	14	14	11	0.824	0.799	0.827	0.806	0.780	0.808	0.331	0.379	0.317
KNUPF107	6	6	6	0.549	0.615	0.721	0.522	0.580	0.683	0.649	0.578	0.439
KNUPF113	12	13	8	0.816	0.827	0.777	0.794	0.809	0.752	0.297	0.318	0.390
Total	144	151	112									
Mean	6.5	6.9	5.1	0.562	0.554	0.578	0.521	0.506	0.533	0.576	0.571	0.541

Group I: Central of South Korea, Group II: Southern of South Korea, Group III: Foreign countries or Unknown.

### Population structure and genetic relationships among 400 accessions of cultivated P. frutescens var. frutescens

**Figure 2:**
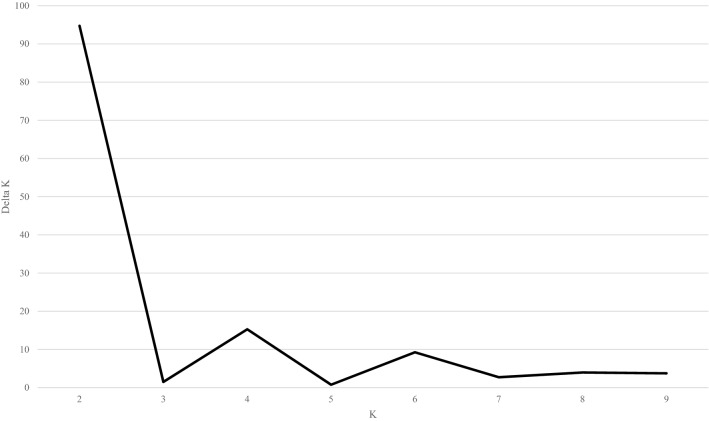
Magnitude of *ΔK* as a function of K. The peak value of ΔK was at *K* = 2 in 400 accessions of cultivated type of var. *frutescens* of *Perilla* crop*.*

**Figure 3:**
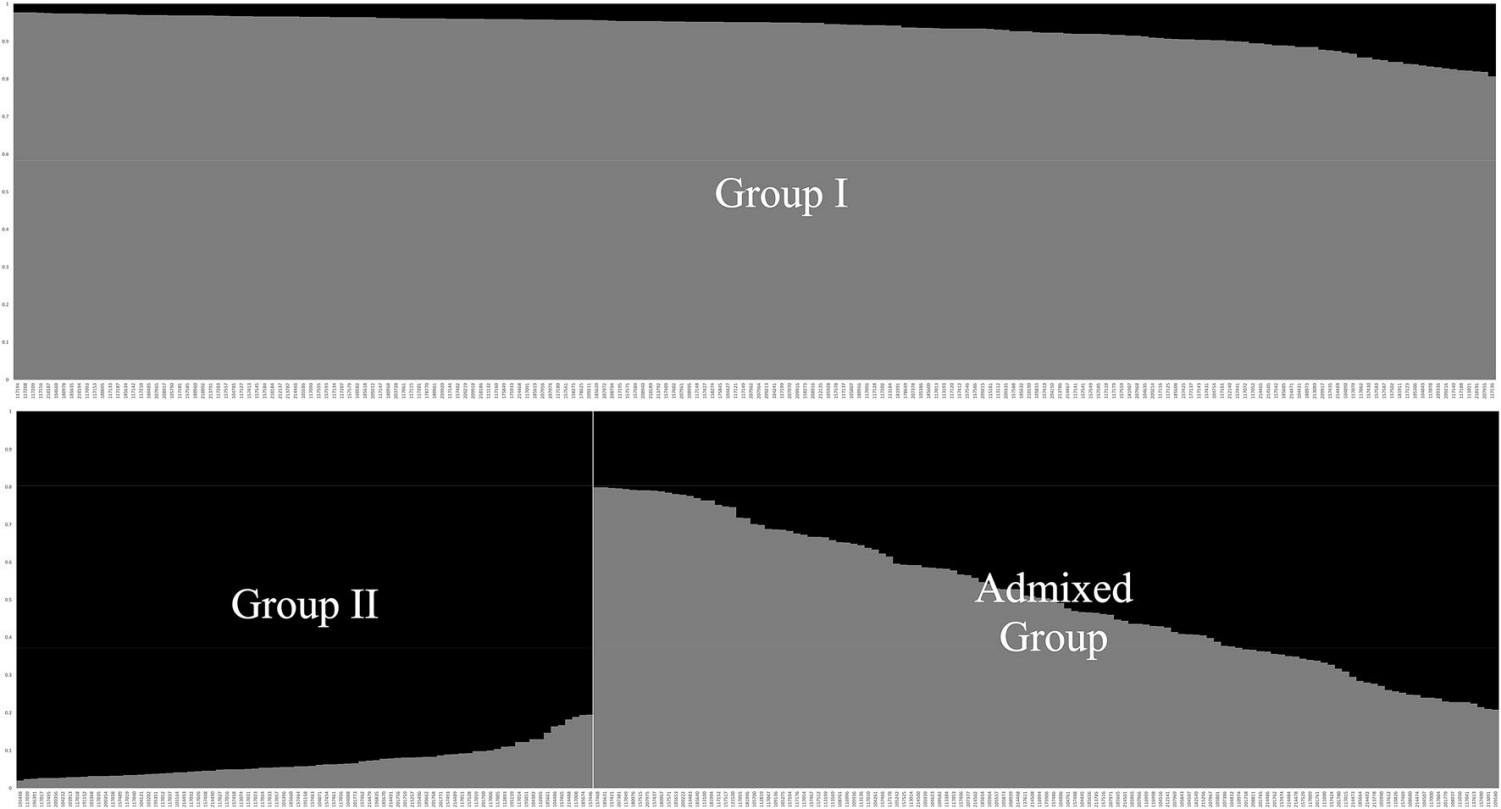
Population structure pattern for the highest *ΔK* value (*K* = 2) of 400 accessions of cultivated type of var. *frutescens* of *Perilla* crop*.*

To understand the population structure among the 400 accessions of cultivated *P*. *frutescens* var. *frutescens*, our study used a model-based approach in STRUCTURE to divide the accessions into subgroups. We applied the ad hoc measure *ΔK* using the method developed by Evanno et al. (2005) to clearly determine the number of subgroups in interpreting the actual *K* values. The highest value of *∆K* for the 400 accessions of cultivated *P*. *frutescens* var. *frutescens* was found at *K* = 2 (Fig. 2). Although we confirmed *K* = 2 by the *∆K* method, some accessions were admixed between these two groups. Thus, we divided the accessions into two main groups and an admixed group in accordance with the method of Wang et al. (2008) based on a threshold of 0.8 (Fig. 3). Based on *K* = 2, Group I included 70 accessions from the central region, 104 accessions from the southern region, and 18 accessions from foreign or unknown. Group II comprised 24 accessions from the central region, 45 accessions from the southern region, and 12 accessions from foreign or unknown. The admixed group included a total of 127 accessions, which consisted of 54 accessions from the central region, 62 accessions from the southern region, and 11 accessions from foreign or unknown.

To confirm the genetic relationships by distance-based analysis among the 400 accessions of cultivated *P*. *frutescens* var. *frutescens*, a dendrogram was constructed using UPGMA. This showed that all *Perilla* accessions were clustered into ten major groups with a genetic similarity of 45.7% (Supplementary Fig. 2). Group IV (182,549, foreign), VI (215,256, central region), VIII (104,486, central region), IX (215,257, central region), and X (214,479, unknown) each included only one accession. Meanwhile, Group I contained 33 accessions (3 accessions from the central region and 30 accessions from the southern region). Group II consisted of 325 accessions (132 accessions from the central region, 167 accessions from the southern region, and 26 accessions from foreign or unknown). Group V and VII included four accessions (3 accessions from the central region and 1 accession from the southern region) and six accessions (1 accession from the central region, 2 accessions from the southern region, and 3 accessions from foreign or unknown), respectively (Supplementary Fig. 2). In addition, Group II, which contained 81.8% of the 400 accessions, was further subdivided into ten sub-clusters with a genetic similarity of 51.8% (Supplementary Fig. 2). The first sub-cluster contained 261 accessions, which divided again into eight sub-groups with a genetic similarity of 56.9% (95 accessions from the central region, 147 accessions from the southern regions, and 19 accessions from foreign or unknown). The second sub-cluster consisted of 13 accessions (10 accession from the central region and 3 accessions from the southern region). The third sub-cluster (2 central and 3 southern region) and fifth sub-cluster (4 central and 1 southern region) each contained five accessions. The fourth sub-cluster consisted of 12 accessions (7 accessions from the central region, 2 accessions from the southern region, and 3 accessions from foreign or unknown). The sixth sub-cluster contained 16 accessions (9 accessions from the central and 7 accessions from the southern region). The seventh sub-cluster (2 accessions from the southern region) and eighth sub-cluster (2 accessions, one collected from the central and one from the southern region) each consisted of two accessions. The ninth sub-cluster included eight accessions (3 accessions from the central region, 1 accession from the southern region, and 4 accessions from foreign or unknown). Finally, the tenth sub-cluster of Group II contained only one accession, collected from the central region (Supplementary Fig. 2).

### Development and evaluation of the core collection

**Figure 4:**
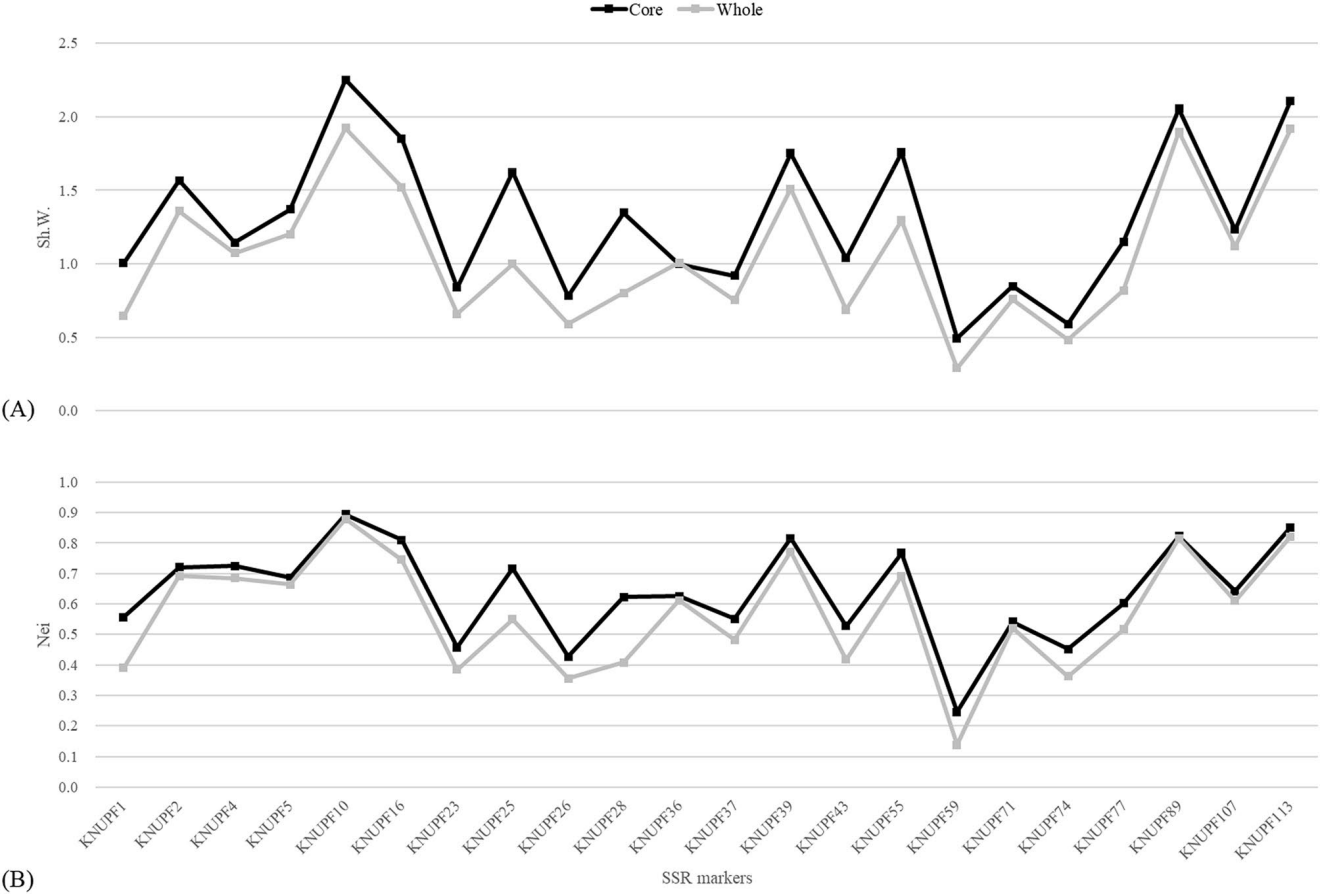
Comparison of a Shannon–Weaver diversity index (**A**) and Nei’s diversity index (**B**) using 22 SSR markers between the core collection and the whole collection.

To establish a core collection, data of 22 SSR genotypes with a total of 173 alleles were used to construct a core set from the 400 accessions of cultivated *P*. *frutescens* var. *frutescens* using PowerCore software^20^. For the core collection, 44 accessions were selected. The core collection contained 16 representatives from the central region, 21 from the southern region, and 7 foreign or unknown (Table 3). The core collection accounted for 11.0% of the whole *Perilla* collection, including 100% of the number of alleles maintained in the whole collection. To compare the allelic richness between the core and whole collections, our study confirmed the diversity by the Shannon–Weaver (Sh.W.) and Nei calculation (Fig. 4, Table 4). The distributions of the Sh.W. and Nei indices in the 22 SSR markers for the core and the whole collections represented a high similarity or those of the core collection were greater than those of the whole collection (Fig. 4, Table 4). The results of a Student’s t test using SPSS software showed that there was no significant difference in the average genetic diversity (Sh.W. and Nei indices) between the core collection and the whole collection (p = 0.102 for Sh.W. and 0.192 for Nei indices). This indicates that the core collection selected herein sufficiently represents allelic richness and genetic diversity of the entire accessions.

**Table 3:** IT number, collection sites, and qualitative traits of core accessions selected by Powercore software.

IT No	Village, town or city	Region	QL1	QL2	QL3	QL4	QL5	QL6	QL7	QL8
117,005	Gyeonggi-do	Suwon-si	1	1	1	3	3	3	1	1
157,458	Gyeonggi-do	Hwaseong-si	3	5	3	3	1	5	3	1
157,474	Gyeonggi-do	Paju-si	-	-	-	-	-	-	-	-
185,618	Gyeonggi-do	Yongin-si	3	1	3	1	5	5	3	1
195,351	Gyeonggi-do	Yeoncheon-gun	5	1	5	3	3	3	3	1
209,212	Gyeonggi-do	Ganghwa-gun	1	3	1	3	1	3	3	1
117,178	Gangwon-do	Chuncheon-si	3	1	3	1	3	3	1	1
137,611	Gangwon-do	Yangyang-gun	5	3	5	5	3	5	7	1
137,612	Gangwon-do	Yangyang-gun	3	3	3	3	3	5	7	1
137,613	Gangwon-do	Yangyang-gun	3	3	3	3	5	5	7	1
157,406	Gangwon-do	Hwacheon-gun	3	3	3	3	3	3	3	2
157,419	Gangwon-do	Pyeongchang-gun	5	3	5	3	1	3	3	1
117,187	Chungcheongbuk-do	Daejeon-si	3	3	3	1	3	5	1	1
180,973	Chungcheongbuk-do	Yeongdong-gun	5	3	5	3	3	5	9	2
214,467	Chungcheongnam-do	Gongju-si	1	1	1	5	3	5	3	1
214,493	Chungcheongnam-do	Gongju-si	5	5	5	3	3	5	3	1
113,569	Gyeongsangbuk-do	Uiseong-gun	5	1	5	1	3	5	3	1
117,023	Gyeongsangbuk-do	Daegu-si	1	3	1	3	3	3	3	1
117,033	Gyeongsangbuk-do	Chilgok-gun	3	3	3	1	1	3	3	1
117,037	Gyeongsangbuk-do	Chilgok-gun	3	3	3	1	1	3	3	1
157,488	Gyeongsangbuk-do	Gyeongju-si	3	3	3	3	3	3	3	1
104,421	Gyeongsangnam-do	Hamyang-gun	-	-	-	-	-	-	-	-
104,445	Gyeongsangnam-do	Hamyang-gun	3	1	3	1	1	3	3	1
104,886	Gyeongsangnam-do	Hamyang-gun	5	3	5	3	1	3	3	1
108,995	Gyeongsangnam-do	Hadong-gun	3	1	3	1	1	3	3	2
185,635	Gyeongsangbuk-do	Mungyeong-si	3	3	3	3	5	5	3	1
181,994	Gyeongsangnam-do	Geochang-gun	3	3	3	5	5	5	3	2
196,835	Gyeongsangnam-do	Hadong-gun	3	3	3	3	3	5	7	1
104,232	Jeollabuk-do	Sunchang-gun	5	3	5	1	3	3	3	2
111,021	Jeollabuk-do	Gochang-gun	3	1	3	3	3	5	3	1
111,080	Jeollabuk-do	Gochang-gun	5	3	5	1	3	3	1	1
117,055	Jeollabuk-do	Gwangju-si	5	1	5	1	1	5	1	1
117,209	Jeollabuk-do	Jinan-gun	5	1	5	1	1	3	3	1
204,150	Jeollabuk-do	Gochang-gun	3	1	3	1	3	3	3	1
157,529	Jeollanam-do	Gurye-gun	3	3	3	3	3	3	5	2
185,654	Jeollanam-do	Gokseong-gun	3	1	3	3	5	3	3	1
117,188	Jeju-do	Jeju-si	3	3	3	1	3	5	5	1
182,549	Foreign	Bhutan	5	1	5	3	7	5	1	1
196,391	Foreign	China	7	5	7	3	3	5	1	1
200,354	Foreign	Nepal	3	1	3	3	7	5	7	1
201,758	Foreign	Japan	3	1	3	1	3	5	7	1
210,184	Unknown	Unknown	3	5	3	1	3	3	1	1
214,479	Unknown	Unknown	3	1	3	5	3	5	3	1
214,489	Unknown	Unknown	5	5	5	1	3	3	3	2

**Table 4:** Comparison of diversity index between 44 core and entire accessions using 22 SSR markers.

SSR loci	C-Allele	C-Sh.W	C-Nei	E-Allele	E-Sh.W	E-Nei
KNUPF1	5	1.003	0.557	5	0.644	0.390
KNUPF2	9	1.567	0.72	9	1.359	0.692
KNUPF4	4	1.145	0.724	4	1.072	0.684
KNUPF5	6	1.369	0.686	6	1.201	0.664
KNUPF10	13	2.252	0.894	13	1.924	0.879
KNUPF16	9	1.852	0.810	9	1.521	0.745
KNUPF23	4	0.840	0.457	4	0.659	0.384
KNUPF25	11	1.623	0.716	11	0.998	0.550
KNUPF26	4	0.783	0.427	4	0.59	0.355
KNUPF28	8	1.348	0.623	8	0.801	0.408
KNUPF36	3	0.998	0.626	3	1.007	0.612
KNUPF37	3	0.918	0.551	3	0.754	0.482
KNUPF39	10	1.753	0.815	10	1.509	0.772
KNUPF43	6	1.038	0.527	6	0.688	0.418
KNUPF55	9	1.757	0.767	9	1.295	0.692
KNUPF59	3	0.493	0.245	3	0.292	0.138
KNUPF71	3	0.847	0.542	3	0.76	0.521
KNUPF74	3	0.589	0.452	3	0.482	0.362
KNUPF77	7	1.149	0.602	7	0.818	0.517
KNUPF89	14	2.056	0.823	14	1.897	0.815
KNUPF107	5	1.232	0.642	5	1.119	0.610
KNUPF113	12	2.109	0.851	12	1.918	0.821
Average	6.9	1.306	0.639	6.9	1.059	0.569

C- core collection group, E- entire group.

Sh.W. and Nei—Diversity index using Shannon & Weaver and Nei calculation by PowerCore software.

### Morphological variation and principal component analysis among 372 accessions of cultivated P. frutescens var. frutescens

The results of examining eight morphological traits of the 372 accessions of cultivated *P*. *frutescens* var. *frutescens* are summarized in Supplementary Table 3.

In the survey of color of leaf surface (QL1), 59 accessions showed light green color, 207 showed green, and 104 showed deep green. The remaining 2 accessions showed light purple color. For color of reverse side leaf (QL2), 144 accessions showed light green color, 203 accessions showed green, and 24 showed deep green. One accession only showed light purple. For stem color (QL3), 58 accessions showed light green, 207 accessions showed green, and 105 accessions showed deep green. Only 2 accessions had light purple. For leaf shape (QL4), 161 accessions showed lanceolate shape, 146 accessions showed heart shape, and 65 accessions showed oblong shape. For degree of pubescence (QL5), 90 accessions showed slightly pubescent, 222 accessions showed normal pubescent, and 58 accessions showed heavily pubescent. The remaining 2 accessions showed more heavily pubescent. In the case of flowering time (QL6), 179 accessions showed intermediate flowering (flowering days from August 15 to September 5) and 193 accessions showed late flowering (flowering days from September 6 after September 25). In the case of the two seed characteristics**,** for seed color (QL7) of the 372 *Perilla* accessions, 86 accessions showed dark brown, 212 accessions showed brown, 24 accessions showed gray, 37 accessions showed white, the remaining 13 accessions showed mixed colors. For seed hardness (QL8) of the 372 *Perilla* accessions, 305 accessions showed soft seeds and the remaining 67 accessions showed hard seeds (Supplement Table 3).

**Figure 5:**
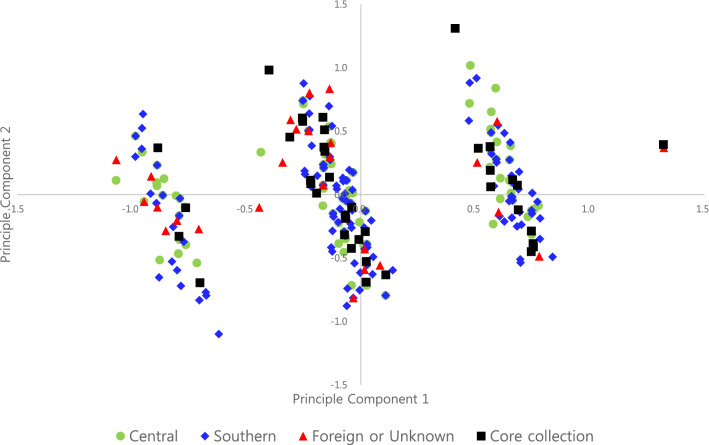
Projection of 372 accessions of cultivated type of var. *frutescens* in the first and second principal components.

This study used this morphological data to perform PCA analysis. The analysis results showed that the first and second principal components accounted for 25.5% and 16.8%, respectively, of the total variance (Table 5). In this study, QL1 and QL3 contributed in the positive direction on the first axis, and QL5 and QL6 contributed in the positive direction on the second axis. Thus, the traits that contributed in the positive direction on the first or second axes are considered to be useful for discrimination between accessions of cultivated *P*. *frutescens* var. *frutescens* (Table 5). Based on the first axis, most accessions of cultivated *P*. *frutescens* var. *frutescens* were clearly separated into three groups on the positive and negative sides of the first axis by color of leaf surface and stem (Fig. 5). Thus, the traits (QL1, QL3, QL5, QL6) that contributed in the positive or negative direction on the first or second axis (Table 5) are considered to be useful for discrimination between accessions of cultivated *P*. *frutescens* var. *frutescens*.

**Table 5:** Cumulative variance of first and second principal components and the loadings of eight qualitative characters on each principal component.

Morphological Character	Eigenvectors	
	1	2
QL5	−0.238	0.774
QL6	−0.194	0.654
QL7	−0.113	−0.204
QL2	−0.002	−0.321
QL4	0.008	0.331
QL8	0.133	−0.132
QL3	0.976	0.155
QL1	0.980	0.154
Cumulative variance (%)	25.5	16.8

In addition, to validate the core set selected by molecular markers, PCA analysis was used to confirm the distribution for the core collection selected by SSR markers. Two accessions (157,474 and 104,421) of the core collection could not be investigated for morphological characteristics (Table 3), but the remaining 42 accessions were evenly distributed in the three parts on a scatter plot by the PCA analysis (Fig. 5).

## Discussion

Information about the genetic diversity of PGRs, which provide useful alleles associated with plant development and improvement, is very important for both the conservation and the utilization of germplasm that has been collected in a genebank^34,35^. With the development of molecular biology, DNA molecular marker technology provides useful information for the analysis of genetic diversity, genetic relationships, population structure, and core collections in the germplasm of many crop species^4,9,16,21–24^.

In the case of *Perilla* crop, much analysis has been performed on genetic diversity, genetic relationships, and population structure using amplified fragment length polymorphisms (AFLP)^29^, random amplification of polymorphic DNAs (RAPD)^26,36^, and SSR markers^37–40^. Unfortunately, and in contrast with other major crop species, other molecular marker technologies in *Perilla* species have not yet been developed. Among these marker systems, as already explained in the Introduction, SSR marker technology is highly polymorphic and reproducible, generally co-dominant and abundant in the plant genome, and it has provided useful information for the analysis of genetic diversity, genetic relationships, population structure, etc. in the germplasm of *Perilla* species^37,41–44^. Recently, SSR primer sets have been developed for *Perilla* crop by many researchers^37,43–46^ and used successfully for the analysis of genetic diversity, genetic relationships, population structure, and association mapping among the accessions of cultivated and weedy types of *Perilla* crop^28,38–40,43,47,48^. Therefore, this study used SSR markers to identify genetic diversity and relationships, population structure, and a core collection of RDA-Genebank *Perilla* germplasm. The GD and PIC values determined in this study of 0.567 and 0.522, respectively (Table 1), were compared with those of previous studies of Ma et al. (2019) and Park et al. (2019) that contained more weedy *Perilla* accessions and showed values of 0.577 and 0.625 for GD and 0.537 and 0.582 for PIC with 21 and 25 SSR markers, respectively. These findings reveal a lower level of genetic diversity in the collection of this study, which mainly consisted of cultivated *P*. *frutescens* var. *frutescens*. Although wild species have not yet been found in *Perilla* crop, many accessions of the weedy type of *Perilla* crop have been reported in East Asia, particularly in South Korea and China, and they show higher genetic diversity than accessions of the cultivated type of *Perilla* crop^25,26,29,38–40,43,49,50^.

Meanwhile, polymorphism of loci can be considered high, medium, or low with GDI > 0.5, GDI < 0.5 and > 0.25, or GDI < 0.25, respectively, according to a report by Vaiman et al. (1994). The population in the current study consisting of the 400 accessions of cultivated *P*. *frutescens* var. *frutescens* has average GD and PIC values of over 0.5 in the 22 SSR markers, indicating that this population has a relatively high genetic diversity (Table 1). Moreover, the 14 (based on GD) and 12 (based on PIC) SSR markers among the entire SSR markers showed a high level of polymorphism based on GDI (each > 0.5) (Table 1). The SSR markers named KNUPF used in this study were recently developed by our previous studies for *Perilla* crop^43,44,46^. Although only cultivated *P*. *frutescens* var. *frutescens* of *Perilla* crop, which has relatively lower diversity, was used as material in this study, many SSR markers showed relatively high GDI^29,38–40,49^. Therefore, these SSR markers were considered useful for identifying genetic diversity and population structure and for selecting a core collection for accessions of *Perilla* crop. Furthermore, these SSR markers will be very useful for genome-wide association study (GWAS) or quantitative traits loci (QTL) analysis, because these markers were developed by using the results of transcriptome analysis for *Perilla* crop^43–45,52^.

In East Asia, although China is considered the origin of *Perilla* crop, South Korea is assumed to be the secondary center of biodiversity of *Perilla* crop because of extensive cultivation, various uses, and high morphological and genetic diversity as well as the existence of weedy types^25,26,49,50^. Recently, cultivated *P*. *frutescens* var. *frutescens* of *Perilla* crop has become a cash crop in South Korea, and the cultivation area has expanded significantly. To maximize the use of genetic resources of cultivated *P*. *frutescens* var. *frutescens* of *Perilla* crop preserved in the RDA-Genebank, the genetic characteristics of the collected resources should be analyzed for efficient conservation and utilization in South Korea. Therefore, this study compared the average allele numbers and GDI values among the central (Group I, 148 accessions) and southern region (Group II, 211 accessions) accessions of South Korea and foreign or unknown (Group III, 41 accessions) accessions (Table 2). The highest allele number was revealed in Group II, followed by Group I and Group III, while the highest GDI values were confirmed in Group III, followed by Group I and Group II (Table 2). Although Group II had the highest allele number, genetic diversity for Group II was lower than that of Group I and II. This result suggests that Group II has the highest number of accessions with more Group II-specific alleles, while this group consists of more accessions with similar genetic characteristics than the other two groups. Moreover, when comparing the South Korea (Group I and II) and foreign accessions (Group III), the South Korea accessions show lower genetic diversity than the foreign accessions (Table 2). This result indicates that the cultivated type of var. *frutescens* of South Korea has a narrower genetic diversity than the foreign accessions, even though South Korea is the secondary center of *Perilla* crop. It may be that the environmental variation in the central and southern regions of South Korea is not severe and that farmers for *Perilla* crop want uniform properties for cultivated type of var. *frutescens*, such as green leaves and seeds with higher oil yield. However, in South Korea, many accessions of weedy type of var. *frutescens* with high genetic and phenotypic diversity were found throughout the region^25,29,38,43,49^.

Although numerous PGRs are currently conserved in genebanks around the world, the large amount of PGRs makes their accessibility and application difficult^3,8^. Moreover, management of these PGRs requires significant effort and expense. It is essential to select and manage a core collection, which can represent the entire collection. The evaluation of genetic distance or population structure among genotypes helps in the selection of parental combinations for generating new segregating populations, which preserves genetic diversity in breeding programs^53^. The identification of the genetic relationships and population structures of an entire collection may provide useful information for core collection selection and the management of PGRs. In this study, to understand the genetic relationships and population structure of 400 accessions of cultivated type of var. *frutescens* from central and southern regions of South Korea, we used two different methods: a model-based approach with STRUCTURE and a distance-based approach with a UPGMA dendrogram (Fig. 3, Supplementary Fig. 2). The STRUCTURE results revealed that the 400 accessions of cultivated var. *frutescens* could be divided into two major groups and an admixed group at *K* = 2 (Figs. 3, 4), while the UPGMA dendrogram results showed that the 400 accessions of cultivated var. *frutescens* were divided into ten major groups with 45.7% of genetic similarity. As mentioned above in the Results section, there was no clear geographical classification by STRUCTURE and UPGMA analysis among the 400 accessions of cultivated var. *frutescens* from the central and southern regions of South Korea and foreign regions (Fig. 3, Supplementary Figs. 1, 2). Xie et al. (2008)^54^ mentioned that population structure and genetic relationship patterns of many accessions are affected by many factors, such as gene flow, selection by environment or human, and breeding systems. *Perilla* crop has a long history of cultivation in East Asia including in South Korea. In South Korea, because many native landraces of *Perilla* crop are still widespread, these seeds might be frequently exchanged between diverse regions by farmers or animals and birds, as previously reported by Lee et al. (2002)^29^.

Meanwhile, because of failure to select a core set by model and distance-based methods, this study utilized PowerCore to construct a core collection with maximum genetic diversity from the entire initial collection and with a minimal number of germplasm resources. In particular, this study used molecular data rather than phenotypic data to construct the core collection. This is because molecular data using molecular markers is more accurate for ensuring genetic diversity of the initial collection and preventing missing data or environmental interactions that typically exist in phenotypic data^55^. PowerCore in this study captured 100% of the alleles with a sampling percentage of 11%, based on 22 SSR markers throughout the collection (Table 4). The percentage of selected samples identified in this study was similar to the percentage (~ 10%) proposed by Brown (1989)^56^, while lower than the suggested percentage (20 ~ 30%) by Yonezawa et al. (1995)^7^. Sh.W. and Nei diversity indices were used for validation of the core collection, and the averages of the Sh.W. index (1.306) and Nei index (0.639) of the *Perilla* core collection were higher than those of the entire initial collection (Sh.W. = 1.059, Nei = 0.569), indicating increased genetic diversity of the core collection. This may be because of the removal of genetic redundancy in the core collection compared with the entire initial collection. It is obvious that this core collection is an exact representation of the diversity of the entire collection (Fig. 4, Table. 4). Although some accessions were absent and only a small number of traits were investigated in the PCA analysis, the accessions of the core collection selected by SSR markers were well reflected in three clusters based on the first axis of the PCA scatter plot by eight morphological traits (Fig. 5). In detail, the morphological analysis revealed all types of color of leaf surface (QL1) and stem color (QL3) in the core collection. Although no accession with light purple for color of reverse side leaf (QL2) was included in the core collection, the remaining three types were contained in the core collection (Supplementary Tables 2, 3). All three types of leaf shape (QL4) were included amongst the 42 accessions in the core collection. Moreover, all four types of degree of pubescence (QL5) were included in the 42 accessions of the core collection. In the case of flowering time (QL6), there were no accessions for early flowering type in the morphological evaluation of the 42 accessions, but the intermediate and late flowering types were contained in the core collection. All five types of seed color (QL7) were included in the 42 accessions of core collection. In addition, most of the 42 accessions in core collection had soft seeds, while seven accessions showed hard seeds (Table 3).

This study constructed the first core collection of Korean *Perilla* accessions and maintained allelic richness. It can be considered as germplasm for identifying useful genes for important agricultural traits. Further modification of the core collection is expected by the continuous addition of new *Perilla* accessions, such as accessions of the two cultivated types of *Perilla* crop and their weedy types. Further analysis of phenotypic and agronomic traits for the core collection is necessary to provide more valuable information for the development and utilization of *Perilla* accessions in breeding programs.

## Materials and methods

### Plant materials and DNA extraction

A total of 400 accessions of cultivated *P*. *frutescens* var. *frutescens* collected in South Korea and other regions (foreign or unknown accessions) were obtained from the RDA-Genebank of the Republic of Korea (http://genebank.rda.go.kr). The accessions of *Perilla* germplasm preserved by the RDA-Genebank of the Republic of Korea have been collected for decades with the approval of farmers for the Korean native *Perilla* accessions. The IT number and location information for these materials is shown in Supplementary Table 1. South Korea is geographically divided into central and southern regions (Supplementary Fig. 1). The central region includes Gangwon-do, Gyeonggi-do, and Chungcheong-do; while the southern region includes Gyeongsang-do, Jeolla-do, and Jeju-do. A total of 359 accessions were collected from the central and southern regions of South Korea, whereas the remaining 41 accessions were either collected from foreign countries (20 accessions) or the collection region is unknown (21 accessions) (Supplementary Table 1). In the central region, 148 accessions were collected from Gangwon-do (46 accessions), Gyeonggi-do (53 accessions), and Chungcheong-do (49 accessions); while in the southern region, 211 accessions were collected from Gyeongsang-do (125 accessions), Jeolla-do (85 accessions), and Jeju-do (1 accession). The accessions used in this study were selected from the RDA-Genebank as breeding materials for the development of leaf vegetable cultivars of cultivated var. *frutescens*. Total DNA was extracted from the young leaf tissue of individual representative plants of each accession according to Plant DNAzol Reagent protocols (GibcoBRL Inc., Grand Island, NY, USA). Our studies were complied with local and national regulations and following Kangwon National University (Chuncheon, Korea) and National Agrobiodiversity Center, National Institute of Agricultural Sciences, RDA (Jeonju, Korea) regulations.

### Evaluation of morphological characteristics

For evaluation of morphological variation among the 400 accessions of cultivated *P*. *frutescens* var. *frutescens,* 20 seeds of each accession were sown in a nursery bed in early May 2020, and kept in a glass house for a month. After that, seven seedlings of each accession were then transplanted to the fields of the experimental farm of Kangwon National University, Chuncheon, Gangwon-do in early June 2020. We examined eight morphological characteristics related to leaf and seed quality and quantity, namely color of leaf surface (QL1), color of reverse side leaf (QL2), stem color (QL3), leaf shape (QL4), degree of pubescence (QL5), flowering time (QL6), seed color (QL7), and seed hardness (QL8) at the appropriate growth stages. These morphological traits, as shown in Supplementary Table 2, were selected based on a previous report by Lee and Ohnishi (2001). In our study, 28 accessions were not used for measurement because of failure of plant growth in the field. Therefore, in this study, only 372 accessions out of the 400 accessions of cultivated *P*. *frutescens* var. *frutescens* were evaluated for morphological characteristics (Supplementary Table 1).

### SSR analysis and DNA electrophoresis

SSR amplification was performed in a total volume of 20 μL containing 20 ng genomic DNA, 1 × polymerase chain reaction (PCR) buffer, 0.2 mM dNTPs, 0.5 μM forward and reverse primers, and 1 unit of *Taq* polymerase (Bio Rad, Hercules, CA). The PCR profile consisted of initial denaturation at 95 °C for 3 min, followed by 36 cycles of 95 °C for 30 s, 55 °C for 30 s, and 72 °C for 1 min 30 s, with a final extension step of 5 min at 72 °C. After PCR, DNA electrophoresis analysis was performed with a QIAxcel advanced system (QIAGEN Co., Hilden, Germany) according to the protocol described in the QIAxcel DNA Handbook. The samples were run in the QIAxcel advanced electrophoresis system, and sample separation was performed over 15 min. Gel images were obtained as the results, and the quantification analysis was performed with QIAxcel software. The results were displayed as gel images and electropherograms acquired from the QIAxcel advanced system software.

### Data analysis

The fragments amplified for each SSR primer set were scored as present (1) or absent (0). Power Marker version 3.25^57^ was used to confirm information on the number of alleles, allele frequency, and genetic diversity index (GDI), such as major allele frequency (MAF), gene diversity (GD), and polymorphic information content (PIC). Genetic similarities (GS) were calculated for each pair of accessions using the Dice similarity index^58^. To illustrate the genetic relationships of the total accessions, a similarity matrix was used to construct an unweighted pair group method with arithmetic mean (UPGMA) dendrogram by the application of SAHN-Clustering from NTSYS-pc V2.1^59^. Principal component analysis (PCA) was performed to detect differences between and within accessions of *P. frutescens* var. *frutescens*. The PCA analysis was performed using NTSYS-pc V2.1^59^. Population structure was investigated for the 400 accessions of cultivated *P*. *frutescens* var. *frutescens* using STRUCTURE 2.2 software^60^. Five independent runs with *K* values ranging from one to ten were performed with 100,000 cycles for both burn-in and the run length. The delta *K* statistic, based on the rate of change in the log probability of data between *K* values^32^, was calculated with STRUCTURE HARVESTER. For extraction of a core collection, this study used PowerCore software, which uses the advanced M strategy implemented through the modified heuristic algorithm for the core collection as described in the user’s manual^20^.

## Supplementary Information

Supplementary Information.
