# Supplementary material for: Construction of a core collection of native Perilla germplasm collected from South Korea based on SSR markers and morphological characteristics
Source: Sci Rep. 2021 Dec 13;11:23891. doi: 10.1038/s41598-021-03362-0 (PMC8668929; doi:10.1038/s41598-021-03362-0)
Supplement: Supplementary file 1 — Supplementary Information. [file 41598_2021_3362_MOESM1_ESM.pdf]

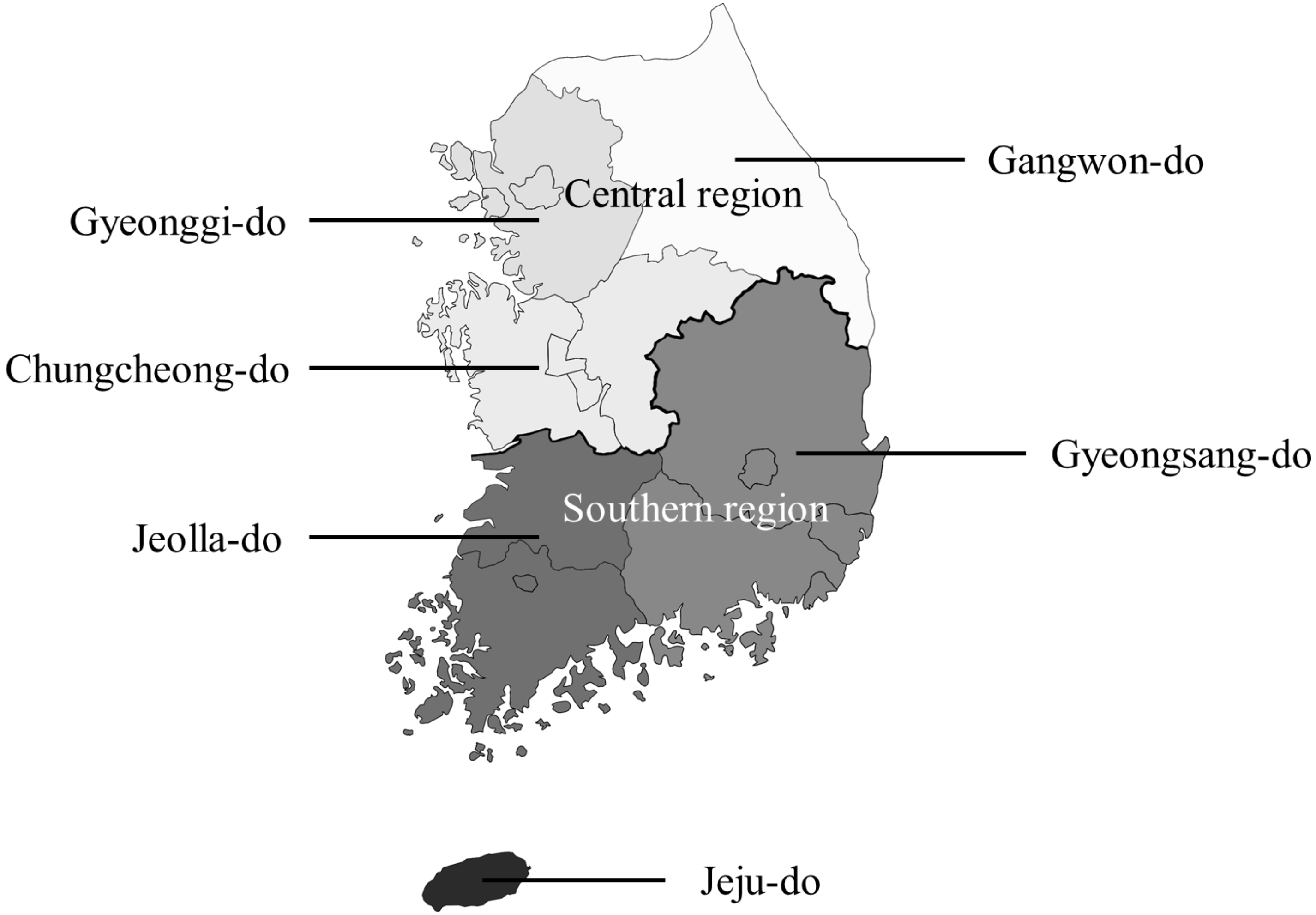

Supplementary Fig. 1. Collection areas of 400 accessions of cultivated type of var. *frutescens* in the central and southern regions of South Korea. The central region (Gangwon-do, Gyeonggi-do, Chungcheong-do) is indicated in light gray. The southern region (Gyeongsang-do, Jeolla-do, Jeju-do) is indicated in dark gray.

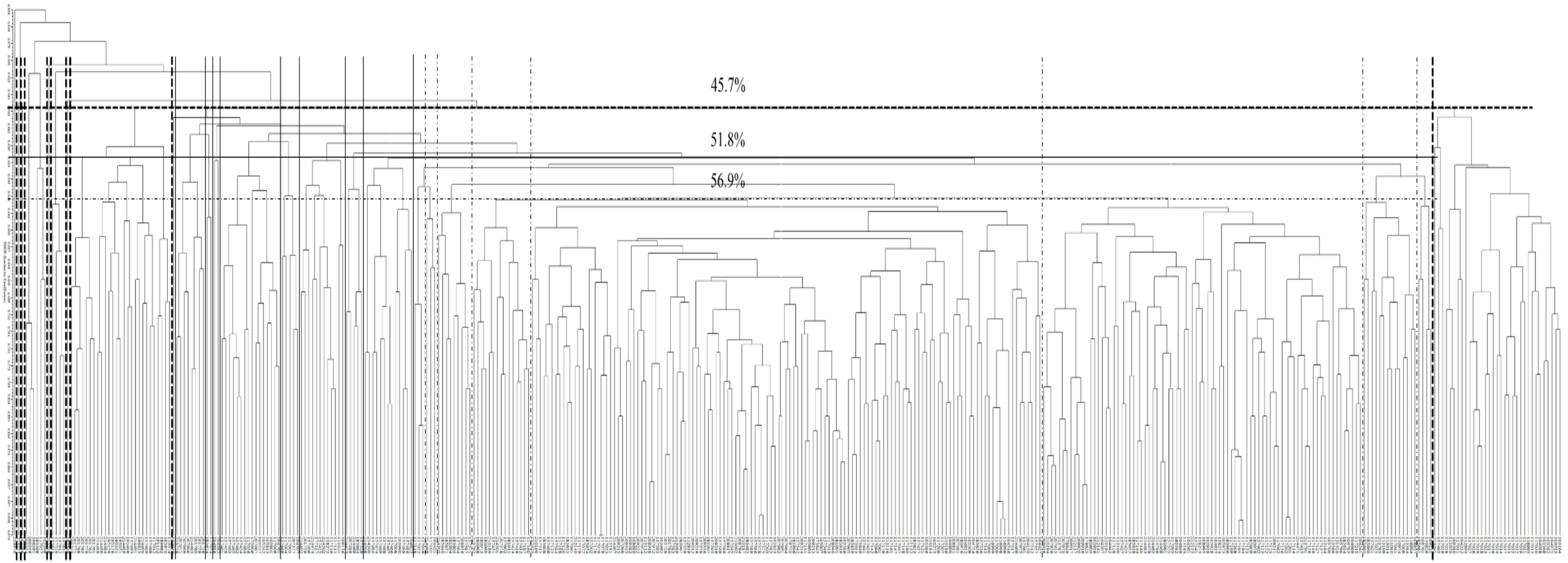

Supplementary Fig. 2. UPGMA dendrogram of 400 accessions of cultivated type of var. *frutescens* based on 22 SSR markers. The dash line represents ten major groups. The solid line represents the sub-clusters of Group II. The dash-dot line represents the separation of the first sub-cluster of Group II.

Supplementary Table 1**.** Collection sites of 400 accessions of cultivated type of var. *frutescens* conserved at the RDA-genebank of South Korea.

IT No.	Village, town or city	Region	IT No.	Village, town or city	Region
110826	Jecheon-si	Chungcheongbuk-do	104486	Jeongseon-gun	Gangwon-do
113112	Jincheon-gun	Chungcheongbuk-do	104507	Pyeongchang-gun	Gangwon-do
113136	Jincheon-gun	Chungcheongbuk-do	104516	Pyeongchang-gun	Gangwon-do
117115	Daejeon-si	Chungcheongbuk-do	104597	Pyeongchang-gun	Gangwon-do
117116	Daejeon-si	Chungcheongbuk-do	104623	Hongcheon-gun	Gangwon-do
117118	Dangjin-si	Chungcheongbuk-do	105306	Chuncheon-si	Gangwon-do
117120	Dangjin-si	Chungcheongbuk-do	105790	Gangneung-si	Gangwon-do
117121	Dangjin-si	Chungcheongbuk-do	117176	Chuncheon-si	Gangwon-do
117123	Cheongju-si	Chungcheongbuk-do	117178	Chuncheon-si	Gangwon-do
117125	Cheongju-si	Chungcheongbuk-do	137611	Yangyang-gun	Gangwon-do
117127	Cheongju-si	Chungcheongbuk-do	137612	Yangyang-gun	Gangwon-do
117128	Cheongju-si	Chungcheongbuk-do	137613	Yangyang-gun	Gangwon-do
117133	Nonsan-si	Chungcheongbuk-do	157402	Chuncheon-si	Gangwon-do
117134	Nonsan-si	Chungcheongbuk-do	157406	Hwacheon-gun	Gangwon-do
117136	Nonsan-si	Chungcheongbuk-do	157409	Chuncheon-si	Gangwon-do
117137	Nonsan-si	Chungcheongbuk-do	157412	Yeongwol-gun	Gangwon-do
117161	Chungju-si	Chungcheongbuk-do	157413	Yeongwol-gun	Gangwon-do
117187	Daejeon-si	Chungcheongbuk-do	157419	Pyeongchang-gun	Gangwon-do
157446	Danyang-gun	Chungcheongbuk-do	157421	Gangneung-si	Gangwon-do
157512	Eumseong-gun	Chungcheongbuk-do	157424	Jeongseon-gun	Gangwon-do
157515	Okcheon-gun	Chungcheongbuk-do	157425	Goseong-gun	Gangwon-do
157549	Jincheon-gun	Chungcheongbuk-do	157427	Goseong-gun	Gangwon-do
180973	Yeongdong-gun	Chungcheongbuk-do	157430	Yangyang-gun	Gangwon-do
185670	Okcheon-gun	Chungcheongbuk-do	157431	Yangyang-gun	Gangwon-do
208811	Jincheon-gun	Chungcheongbuk-do	157435	Yangyang-gun	Gangwon-do
214473	Okcheon-gun	Chungcheongbuk-do	157437	Yangyang-gun	Gangwon-do
214484	Jecheon-si	Chungcheongbuk-do	157441	Wonju-si	Gangwon-do
185674	Jecheon-si	Chungcheongbuk-do	158273	Yeongwol-gun	Gangwon-do
105964	Yesan-gun	Chungcheongnam-do	158274	Yeongwol-gun	Gangwon-do
105965	Yesan-gun	Chungcheongnam-do	158275	Yeongwol-gun	Gangwon-do
157571	Buyeo-gun	Chungcheongnam-do	185605	Yangyang-gun	Gangwon-do
157578	Asan-si	Chungcheongnam-do	185608	Wonju-si	Gangwon-do
157579	Asan-si	Chungcheongnam-do	185609	Wonju-si	Gangwon-do
185664	Asan-si	Chungcheongnam-do	189662	Pyeongchang-gun	Gangwon-do
185665	Yesan-gun	Chungcheongnam-do	195051	Chuncheon-si	Gangwon-do
207961	Cheongyang-gun	Chungcheongnam-do	195496	Yanggu-gun	Gangwon-do
207962	Hongseong-gun	Chungcheongnam-do	195498	Hwacheon-gun	Gangwon-do
207964	Hongseong-gun	Chungcheongnam-do	212140	Yangyang-gun	Gangwon-do
207965	Boryeong-si	Chungcheongnam-do	212141	Goseong-gun	Gangwon-do
207966	Seocheon-gun	Chungcheongnam-do	214500	Yangyang-gun	Gangwon-do
207967	Seocheon-gun	Chungcheongnam-do	214501	Yangyang-gun	Gangwon-do
207968	Nonsan-si	Chungcheongnam-do	214502	Yangyang-gun	Gangwon-do
207969	Geumsan-gun	Chungcheongnam-do	214508	Cheorwon-gun	Gangwon-do
208794	Asan-si	Chungcheongnam-do	215256	Cheorwon-gun	Gangwon-do
214466	Yeongi-gun	Chungcheongnam-do	215257	Cheorwon-gun	Gangwon-do
214467	Gongju-si	Chungcheongnam-do	105833	Hwaseong-si	Gyeonggi-do
214492	Daejeon-si	Chungcheongnam-do	112819	Hwaseong-si	Gyeonggi-do
214493	Gongju-si	Chungcheongnam-do	112893	Paju-si	Gyeonggi-do
157575	Seocheon-gu	Chungcheongnam-do	113100	Suwon-si	Gyeonggi-do
103282	Samcheok-shi	Gangwon-do	116994	Hwaseong-si	Gyeonggi-do

Supplementary Table 1. Continued

IT No.	Village, town or city	Region	IT No.	Village, town or city	Region
116995	Hwaseong-si	Gyeonggi-do	104761	Gimcheon-si	Gyeongsangbuk-do
116996	Hwaseong-si	Gyeonggi-do	104785	Gimcheon-si	Gyeongsangbuk-do
116998	Hwaseong-si	Gyeonggi-do	105557	Gimcheon-si	Gyeongsangbuk-do
116999	Hwaseong-si	Gyeonggi-do	105801	Andong-si	Gyeongsangbuk-do
117000	Hwaseong-si	Gyeonggi-do	108680	Yecheon-gun	Gyeongsangbuk-do
117004	Suwon-si	Gyeonggi-do	108761	Yecheon-gun	Gyeongsangbuk-do
117005	Suwon-si	Gyeonggi-do	110974	Uiseong-gun	Gyeongsangbuk-do
117006	Suwon-si	Gyeonggi-do	113199	Gimcheon-si	Gyeongsangbuk-do
117008	Suwon-si	Gyeonggi-do	113569	Uiseong-gun	Gyeongsangbuk-do
117009	Suwon-si	Gyeonggi-do	115181	Seongju-gun	Gyeongsangbuk-do
117010	Suwon-si	Gyeonggi-do	117011	Daegu-si	Gyeongsangbuk-do
117096	Paju-si	Gyeonggi-do	117013	Daegu-si	Gyeongsangbuk-do
117203	Yongin-si	Gyeonggi-do	117014	Daegu-si	Gyeongsangbuk-do
117207	Suwon-si	Gyeonggi-do	117015	Daegu-si	Gyeongsangbuk-do
157454	Hwaseong-si	Gyeonggi-do	117016	Daegu-si	Gyeongsangbuk-do
157458	Hwaseong-si	Gyeonggi-do	117017	Daegu-si	Gyeongsangbuk-do
157461	Hwaseong-si	Gyeonggi-do	117018	Daegu-si	Gyeongsangbuk-do
157462	Hwaseong-si	Gyeonggi-do	117019	Daegu-si	Gyeongsangbuk-do
157463	Hwaseong-si	Gyeonggi-do	117023	Daegu-si	Gyeongsangbuk-do
157464	Hwaseong-si	Gyeonggi-do	117024	Daegu-si	Gyeongsangbuk-do
157465	Hwaseong-si	Gyeonggi-do	117026	Daegu-si	Gyeongsangbuk-do
157466	Hwaseong-si	Gyeonggi-do	117027	Chilgok-gun	Gyeongsangbuk-do
157467	Hwaseong-si	Gyeonggi-do	117031	Chilgok-gun	Gyeongsangbuk-do
157468	Hwaseong-si	Gyeonggi-do	117032	Chilgok-gun	Gyeongsangbuk-do
157469	Hwaseong-si	Gyeonggi-do	117033	Chilgok-gun	Gyeongsangbuk-do
157474	Paju-si	Gyeonggi-do	117035	Chilgok-gun	Gyeongsangbuk-do
157480	Yangpyeong-gun	Gyeonggi-do	117037	Chilgok-gun	Gyeongsangbuk-do
157555	Suwon-si	Gyeonggi-do	117038	Chilgok-gun	Gyeongsangbuk-do
157557	Suwon-si	Gyeonggi-do	117039	Chilgok-gun	Gyeongsangbuk-do
157559	Suwon-si	Gyeonggi-do	117040	Chilgok-gun	Gyeongsangbuk-do
185618	Yongin-si	Gyeonggi-do	117041	Chilgok-gun	Gyeongsangbuk-do
195351	Yeoncheon-gun	Gyeonggi-do	117047	Pohang-si	Gyeongsangbuk-do
207975	Ganghwa-gun	Gyeonggi-do	117049	Pohang-si	Gyeongsangbuk-do
208916	Ongjin-gun	Gyeonggi-do	117072	Andong-si	Gyeongsangbuk-do
208917	Ongjin-gun	Gyeonggi-do	117078	Chilgok-gun	Gyeongsangbuk-do
209211	Ganghwa-gun	Gyeonggi-do	117079	Andong-si	Gyeongsangbuk-do
209212	Ganghwa-gun	Gyeonggi-do	117090	Yeongyang-gun	Gyeongsangbuk-do
209213	Ganghwa-gun	Gyeonggi-do	117091	Yeongyang-gun	Gyeongsangbuk-do
209214	Ganghwa-gun	Gyeonggi-do	117093	Yeongyang-gun	Gyeongsangbuk-do
209215	Ganghwa-gun	Gyeonggi-do	117101	Yecheon-gun	Gyeongsangbuk-do
209216	Ganghwa-gun	Gyeonggi-do	117143	Daegu-si	Gyeongsangbuk-do
209219	Ganghwa-gun	Gyeonggi-do	117144	Daegu-si	Gyeongsangbuk-do
209222	Ganghwa-gun	Gyeonggi-do	117152	Uljin-gun	Gyeongsangbuk-do
209335	Ganghwa-gun	Gyeonggi-do	117153	Andong-si	Gyeongsangbuk-do
209336	Ganghwa-gun	Gyeonggi-do	117160	Uiseong-gun	Gyeongsangbuk-do
213785	Pyeongtaek-si	Gyeonggi-do	117195	Daegu-si	Gyeongsangbuk-do
214471	suwon42	Gyeonggi-do	117201	Uljin-gun	Gyeongsangbuk-do
207974	Ganghwa-gun	Gyeonggi-do	117202	Yeongyang-gun	Gyeongsangbuk-do
104635	Seongju-gun	Gyeongsangbuk-do	121520	Sangju-si	Gyeongsangbuk-do
104669	Seongju-gun	Gyeongsangbuk-do	105807	Andong-si	Gyeongsangbuk-do

Supplementary Table 1. Continued

IT No.	Village, town or city	Region	IT No.	Village, town or city	Region
113184	Andong-si	Gyeongsangbuk-do	117156	Changwon-si	Gyeongsangnam-do
113193	Gimcheon-si	Gyeongsangbuk-do	157516	Geochang-gun	Gyeongsangnam-do
117147	Chilgok-gun	Gyeongsangbuk-do	157517	Geochang-gun	Gyeongsangnam-do
157486	Chilgok-gun	Gyeongsangbuk-do	157525	Yangsan-si	Gyeongsangnam-do
157488	Gyeongju-si	Gyeongsangbuk-do	157528	Hadong-gun	Gyeongsangnam-do
113074	Seongju-gun	Gyeongsangbuk-do	181994	Geochang-gun	Gyeongsangnam-do
117012	Daegu-si	Gyeongsangbuk-do	181996	Geochang-gun	Gyeongsangnam-do
157489	Gyeongju-si	Gyeongsangbuk-do	185619	Miryang-si	Gyeongsangnam-do
157493	Gyeongju-si	Gyeongsangbuk-do	185631	Hamyang-gun	Gyeongsangnam-do
157495	Uljin-gun	Gyeongsangbuk-do	185632	Hamyang-gun	Gyeongsangnam-do
157498	Yeongyang-gun	Gyeongsangbuk-do	196835	Hadong-gun	Gyeongsangnam-do
175845	Cheongsong-gun	Gyeongsangbuk-do	207955	Naju-si	Gyeongsangnam-do
175849	Yeongyang-gun	Gyeongsangbuk-do	207956	Jinju-si	Gyeongsangnam-do
175906	Bonghwa-gun	Gyeongsangbuk-do	209916	Sacheon-si	Gyeongsangnam-do
175933	Bonghwa-gun	Gyeongsangbuk-do	209917	Sacheon-si	Gyeongsangnam-do
180960	Sangju-si	Gyeongsangbuk-do	209918	Jinju-si	Gyeongsangnam-do
180961	Sangju-si	Gyeongsangbuk-do	209920	Sancheong-gun	Gyeongsangnam-do
180966	Gimcheon-si	Gyeongsangbuk-do	212137	Goseong-gun	Gyeongsangnam-do
180968	Gimcheon-si	Gyeongsangbuk-do	213791	Tongyeong-si	Gyeongsangnam-do
181991	Gimcheon-si	Gyeongsangbuk-do	214469	Gimhae-si	Gyeongsangnam-do
185634	Mungyeong-si	Gyeongsangbuk-do	214498	Goseong-gun	Gyeongsangnam-do
185635	Mungyeong-si	Gyeongsangbuk-do	214505	Milyang10	Gyeongsangnam-do
185639	Andong-si	Gyeongsangbuk-do	210194	Sacheon-si	Gyeongsangnam-do
185640	Uljin-gun	Gyeongsangbuk-do	104232	Sunchang-gun	Jeollabuk-do
191152	Seongju-gun	Gyeongsangbuk-do	104261	Namwon-si	Jeollabuk-do
203728	Bonghwa-gun	Gyeongsangbuk-do	104382	Namwon-si	Jeollabuk-do
207377	Gyeongju-si	Gyeongsangbuk-do	104849	Gunsa-si	Jeollabuk-do
207378	Yeongcheon-si	Gyeongsangbuk-do	105242	Imsil-gun	Jeollabuk-do
207381	Cheongdo-gun	Gyeongsangbuk-do	105275	Imsil-gun	Jeollabuk-do
207386	Cheongdo-gun	Gyeongsangbuk-do	105386	Namwon-si	Jeollabuk-do
210092	Andong-si	Gyeongsangbuk-do	105700	Jangsu-gun	Jeollabuk-do
214483	Andong-si	Gyeongsangbuk-do	105843	Imsil-gun-	Jeollabuk-do
157502	Uiseong-gun	Gyeongsangbuk-do	105873	Namwon-si	Jeollabuk-do
180967	Gimcheon-si	Gyeongsangbuk-do	109136	Jinan-gun	Jeollabuk-do
103164	Ulsan-si	Gyeongsangnam-do	111021	Gochang-gun	Jeollabuk-do
104071	Sacheon-si	Gyeongsangnam-do	111050	Gochang-gun	Jeollabuk-do
104088	Gimcheon-si	Gyeongsangnam-do	111080	Gochang-gun	Jeollabuk-do
104121	Gimcheon-si	Gyeongsangnam-do	113014	Jeongeup-si	Jeollabuk-do
104241	Hadong-gun	Gyeongsangnam-do	113384	Gochang-gun	Jeollabuk-do
104421	Hamyang-gun	Gyeongsangnam-do	117052	Gwangju-si	Jeollabuk-do
104427	Hadong-gun	Gyeongsangnam-do	117053	Gwangju-si	Jeollabuk-do
104445	Hamyang-gun	Gyeongsangnam-do	117054	Gwangju-si	Jeollabuk-do
104754	Hadong-gun	Gyeongsangnam-do	117055	Gwangju-si	Jeollabuk-do
104832	Hadong-gun	Gyeongsangnam-do	117056	Gwangju-si	Jeollabuk-do
104886	Hamyang-gun	Gyeongsangnam-do	117059	Namwon-si	Jeollabuk-do
104890	Goseong-gun	Gyeongsangnam-do	117061	Namwon-si	Jeollabuk-do
105928	Hadong-gun	Gyeongsangnam-do	117062	Sunchang-gun	Jeollabuk-do
105936	Hadong-gun	Gyeongsangnam-do	117179	Gwangju-si	Jeollabuk-do
105939	Masan-si	Gyeongsangnam-do	117180	Gwangju-si	Jeollabuk-do
108995	Hadong-gun	Gyeongsangnam-do	117208	Jinan-gun	Jeollabuk-do

Supplementary Table 1. Continued

IT No.	Village, town or city	Region	IT No.	Village, town or city	Region
117209	Jinan-gun	Jeollabuk-do	213089	Sinan-gun	Jeollanam-do
108937	Muju-gun	Jeollabuk-do	213091	Wando-gun	Jeollanam-do
105396	Imsil-gun	Jeollabuk-do	213792	Wando-gun	Jeollanam-do
157583	Namwon-si	Jeollabuk-do	214465	Hampyeong-gun	Jeollanam-do
157584	Namwon-si	Jeollabuk-do	214490	Goheung-gun	Jeollanam-do
157585	Namwon-si	Jeollabuk-do	214491	Goheung-gun	Jeollanam-do
157586	Namwon-si	Jeollabuk-do	207972	Boseong-gun	Jeollanam-do
157587	Gochang-gun	Jeollabuk-do	214468	Sinan-gun	Jeollanam-do
157588	Gochang-gun	Jeollabuk-do	117188	Jeju-si	Jeju-do
157589	Gochang-gun	Jeollabuk-do	117140	Japan	Foreign
157593	Gimje-si	Jeollabuk-do	117141	Japan	Foreign
157594	Gimje-si	Jeollabuk-do	117142	Japan	Foreign
157595	Gimje-si	Jeollabuk-do	182549	Bhutan	Foreign
157602	Gimje-si	Jeollabuk-do	196391	China	Foreign
180485	Gochang-gun	Jeollabuk-do	200354	Nepal	Foreign
180605	Jinan-gun	Jeollabuk-do	200356	Nepal	Foreign
180976	Muju-gun	Jeollabuk-do	201756	Japan	Foreign
180978	Muju-gun	Jeollabuk-do	201758	Japan	Foreign
181907	Gochang-gun	Jeollabuk-do	201759	Japan	Foreign
185656	Namwon-si	Jeollabuk-do	201760	Japan	Foreign
185660	Jeonju-si	Jeollabuk-do	201761	Japan	Foreign
185661	Jeongeup-si	Jeollabuk-do	201762	Japan	Foreign
195158	Namwon-si	Jeollabuk-do	201765	Japan	Foreign
195159	Namwon-si	Jeollabuk-do	201768	Japan	Foreign
204150	Gochang-gun	Jeollabuk-do	201771	Japan	Foreign
208939	Gunsan-si	Jeollabuk-do	201773	Japan	Foreign
208940	Gunsan-si	Jeollabuk-do	201779	Japan	Foreign
181911	Gochang-gun	Jeollabuk-do	201780	Japan	Foreign
103368	Boseong-gun	Jeollanam-do	213786	United States	Foreign
103913	Namwon-si	Jeollanam-do	117194	unknown	Unknown
105990	Gokseong-gun	Jeollanam-do	117199	unknown	Unknown
109580	Boseong-gun	Jeollanam-do	117210	unknown	Unknown
109582	Boseong-gun	Jeollanam-do	177137	Unknown	Unknown
111132	Hamyang-gun	Jeollanam-do	178625	Unknown	Unknown
117148	Namwon-si	Jeollanam-do	178649	Unknown	Unknown
117149	Namwon-si	Jeollanam-do	178770	Unknown	Unknown
105586	Boseong-gun	Jeollanam-do	210184	Unknown	Unknown
117057	Gwangju-si	Jeollanam-do	210186	Unknown	Unknown
157529	Gurye-gun	Jeollanam-do	210187	Unknown	Unknown
157541	Jangseong-gun	Jeollanam-do	210189	Unknown	Unknown
157542	Jangseong-gun	Jeollanam-do	210190	Unknown	Unknown
157545	Damyang-gun	Jeollanam-do	210191	Unknown	Unknown
157546	Damyang-gun	Jeollanam-do	213778	Unknown	Unknown
157561	Hampyeong-gun	Jeollanam-do	213787	Unknown	Unknown
160582	Boseong-gun	Jeollanam-do	214474	Unknown	Unknown
185653	Jangseong-gun	Jeollanam-do	214478	Unknown	Unknown
185654	Gokseong-gun	Jeollanam-do	214479	Unknown	Unknown
207970	Suncheon-si	Jeollanam-do	214489	Unknown	Unknown
207971	Suncheon-si	Jeollanam-do	214486	Unknown	Unknown
212135	Jangseong-gun	Jeollanam-do	214488	Unknown	Unknown

Supplementary Table 2. Characters used in the morphological analysis of the 372 accessions of cultivated var. *frutescens*.

Morphological character		Category
QL1	Color of leaf surface	1-light green, 3-green, 5-deep green, 7-light purple
QL2	Color of reverse side leaf	1-light green, 3-green, 5-deep green, 7-light purple
QL3	Stem color	1-light green, 3-green, 5-deep green, 7-light purple
QL4	Leaf shape	1-lanceolate, 3-heart shape, 5-oblong
QL5	Degree of pubescence	1-slightly pubescent, 3-normal pubescent, 5-heavily pubescent, 7-more heavily pubescent
QL6	Flowering time	1-early, 3-intermediate, 5-late
QL7	Seed color	1-dark brown, 3-brown, 5-gray, 7-white, 9-mixed
QL8	Seed hardness	1-soft, 2-hard

Supplementary Table 3. Morphological variations of collection area of 372 accessions of var. *frutescens* for eight qualitative traits.

Morphological Character	Central Region (N=138)	Southern Region (N=196)	Foreign region or Unknown (N=38)	Total accessions (N=372)
QL1	1 (26*), 3 (73), 5 (39)	1 (24), 3 (115), 5 (57)	1 (9), 3 (19), 5 (8), 7 (2)	1 (59), 3 (207), 5 (104), 7 (2)
QL2	1 (52), 3 (73), 5 (13)	1 (80), 3 (109), 5 (7)	1 (12), 3 (21), 5 (4), 7 (1)	1 (144), 3 (203), 5 (24), 7 (1)
QL3	1 (27), 3 (72), 5 (39)	1 (24), 3 (115), 5 (57)	1 (7), 3 (20), 5 (9), 7 (2)	1 (58), 3 (207), 5 (105), 7 (2)
QL4	1 (45), 3 (64), 5 (29)	1 (97), 3 (70), 5 (29)	1 (19), 3 (12), 5 (7)	1 (161), 3 (146), 5 (65)
QL5	1 (22), 3 (96), 5 (20)	1 (62), 3 (105), 5 (29)	1 (6), 3 (21), 5 (9), 7 (2)	1 (90), 3 (222), 5 (58), 7 (2)
QL6	3 (69), 5 (69)	3 (97), 5 (99)	3 (13), 5 (25)	3 (179), 5 (193)
QL7	1 (28), 3 (93), 5 (4), 7 (9), 9 (4)	1 (45), 3 (109), 5 (16), 7 (19), 9 (7)	1 (13), 3 (10), 5 (4), 7 (9), 9 (2)	1 (86), 3 (212), 5 (24), 7 (37), 9 (13)
QL8	1 (113), 2 (25)	1 (162), 2 (34)	1 (30), 2 (8)	1 (305), 2 (67)

* The number of individuals in the 372 accessions of var. *frutescens* for each trait type.
